# Isolated Palsy of the Cisternal Segment of the Hypoglossal Nerve Due to Arterial Dissection of the V4 Segment of the Vertebral Artery: A Case Report With a Side Note on Nerve Trunk Ischemia

**DOI:** 10.7759/cureus.9930

**Published:** 2020-08-21

**Authors:** Hassan Kesserwani

**Affiliations:** 1 Neurology, Flowers Medical Group, Dothan, USA

**Keywords:** vertebral arteries, arterial dissection

## Abstract

We describe the case of a 57-year-old woman who developed severe left-sided neck pain followed by difficulty chewing, oropharyngeal dysphagia, and dysarthria. Clinically, she developed an isolated left-sided deviation of the tongue with protrusion; an isolated cisternal hypoglossal nerve palsy. A magnetic resonance angiogram imaging study revealed a left V4 cisternal vertebral artery dissection with normal diffusion-weighted imaging of the lower medulla. We outline the anatomy, histology, and pathophysiology of ischemic injury of nerve trunks and briefly review the recent trials of vertebral and carotid artery dissections.

## Introduction

The twelfth cranial nerve, the hypoglossal nerve, emerges as a series of rootlets in the medulla between the pyramid and inferior olive. The rootlets fuse into two roots before they enter the hypoglossal canal. This segment between the medulla and hypoglossal canal is known as the cisternal segment. In the pre-medullary cistern, the V4 segment of the vertebral artery lies immediately behind it. The vertebral artery is divided into four segments: V1 from its origin at the subclavian artery to the transverse foramen of C6, V2 from the transverse foramen of C6 to C2, V3 from the transverse foramen of C2 and its entry into the dura, and finally V4 from its dural entry into the confluence with the contralateral vertebral artery to form the basilar artery. Here in the subarachnoid space, the V4 segment of the vertebral artery lies in close proximity to the hypoglossal roots, lying immediately behind it. Gibo et al. flesh out the anatomy of the cisternal segment of the hypoglossal nerve and its blood supply in great detail [1].

The hypoglossal nerve is purely motor, supplying the intrinsic and extrinsic muscles of the tongue. Damage to the nerve disrupts the balancing action of the genioglossus, which deviates the tongue to the contralateral side, hence leading to protrusion of the tongue towards the paretic nerve. Damage to the hypoglossal nerve leads to the classic triad of difficulty chewing, dysphagia, and dysarthria.

Injury to the cisternal segment of the hypoglossal nerve can arise intrinsically from vertebral artery dissection, dolichoectasia of the vertebral artery, or from a schwannoma of the hypoglossal nerve [2,3,4]. Extrinsically, damage may arise from trauma and invasive malignancy [5]. The hypoglossal nerve derives part of its blood supply from the vertebral artery. Injury to the nerve can arise from ischemia or direct compression.

We describe the rare case of a healthy 57-year-old woman who developed severe left-sided neck pain followed by difficulty manipulating food in her mouth, dysarthria, difficulty swallowing, and deviation of the tongue to the side of the lesion, with protrusion. A magnetic resonance angiography (MRA) imaging study revealed a left distal V4 vertebral artery dissection. The patient was treated with baby aspirin, 81 milligrams (mg) daily and her symptoms resolved within a few weeks. In the discussion segment, we review the pathophysiology of injury to peripheral nerves in general and postulate about potential mechanisms of injury to the hypoglossal nerve from vertebral artery dissection. Furthermore, we outline in simple tabular form the studies that confer the bio-equivalency of anti-platelet therapy and anti-coagulation therapy with warfarin in vertebral and carotid artery dissections.

## Case presentation

Our patient is a fit and healthy 57-year-old woman who woke up one morning with severe left-sided neck pain and stiffness. One day later she noted that her speech was a little thick. She developed difficulty manipulating food in her mouth and initiating the swallow of a morsel of food. Upon inspection of her mouth and throat, she noted that her tongue deviated to the left upon protrusion. By then, the neck pain had subsided significantly. She denied any diplopia, facial, or oropharyngeal numbness. She denied any numbness or weakness of the limbs. She was also free of vertigo or imbalance. She also denied any autonomic symptoms such as anhidrosis, hyperhidrosis, flushing of the skin, or abnormal perception of temperature such as localized warm or cold sensations or abnormal feel for cold or warm objects. She denied any trauma to the head or neck, denied any recent chiropractic manipulation or prior history of arterial dissection.

Her past medical history was significant for hyperlipidemia. Her family history was negative for premature coronary artery or cerebrovascular disease. Her medications included atorvastatin and estrogen replacement therapy. She was a non-smoker and she did not drink alcohol.

Her blood pressure (BP) was 156/59 with a pulse of 79. Her weight was 148 pounds, height 5 foot 3 inches with a body-mass index (BMI) of 26.2. The precordial examination revealed no murmurs and carotid artery auscultation was devoid of any bruits. Her gait including cadence and tandem was entirely normal. Her speech was notably thick but without hypophonia or aphasia. Prosody was intact. Her cranial nerve examination was entirely normal except for motor tongue testing which revealed deviation of the tongue to the left when protruded. One should highlight the absence of spontaneous or gaze-evoked nystagmus. Accommodation was active with the absence of a Horner's pupil. It should also be mentioned that her gag was active and symmetric with preserved pharyngeal sensation. Shoulder shrug was symmetric and sternocleidomastoid torsional action was also symmetric. Finger to nose and heel to shin action was symmetric and smooth. No intention tremor or clumsiness was noted. Power was entirely normal and symmetric in both the upper and lower extremities. Deep tendon reflexes were lively and symmetric. Sensory examination failed to reveal any crossed anesthesia to pin prick or cold temperature sensation, by specifically testing facial and appendicular sensation. A deliberate skin examination failed to reveal any flushed or hyperemic skin.

**Figure 1:**
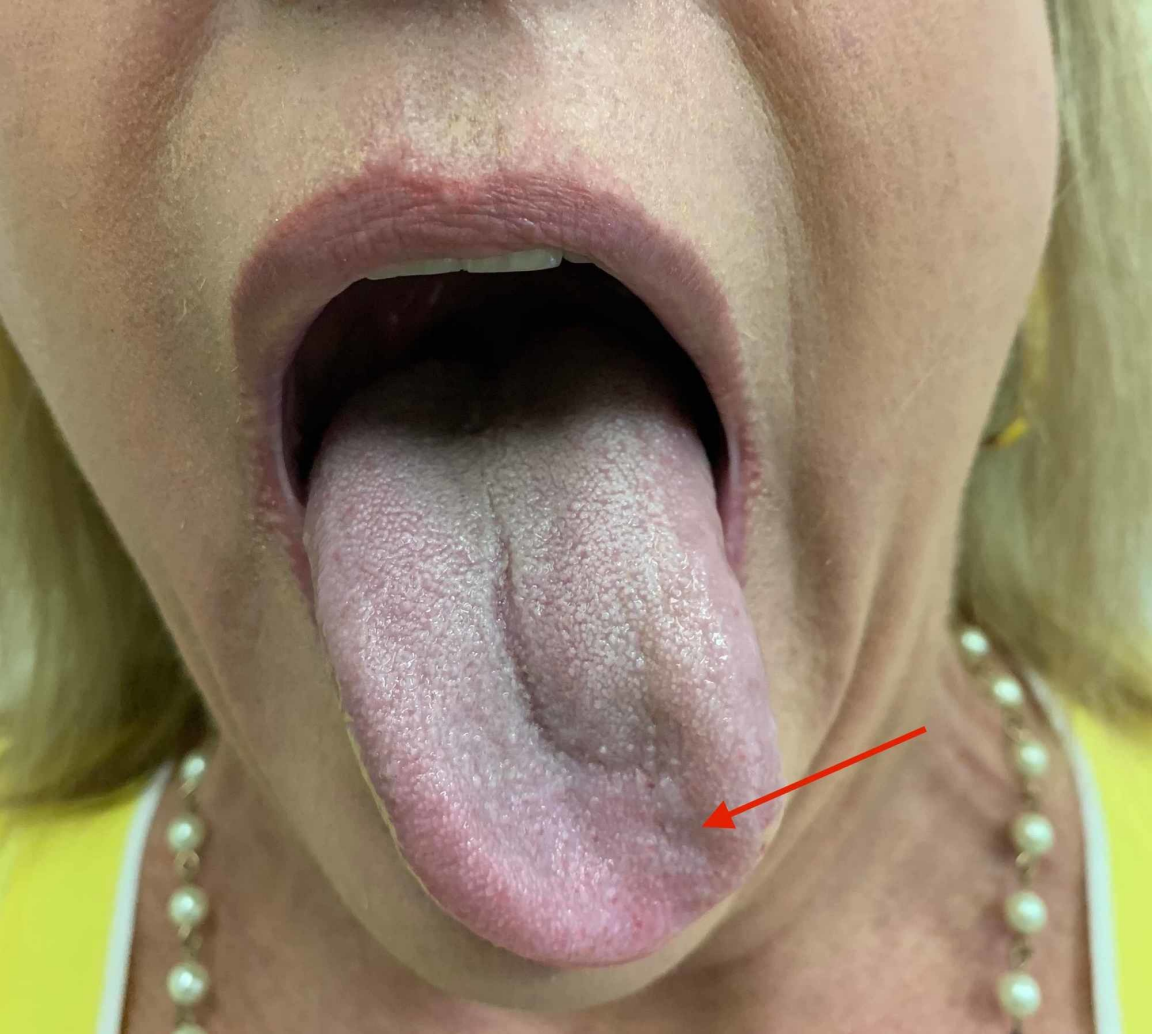
Photograph. Tongue deviation to the left upon protrusion: note corrugation on surface of tongue likely due to muscle atony (red arrow)

It can be safely said that the patient failed to reveal any of the clinical manifestations of the Wallenberg syndrome, and we can accurately state that the only localized finding was an isolated left hypoglossal nerve palsy (Figure 1).

**Figure 2:**
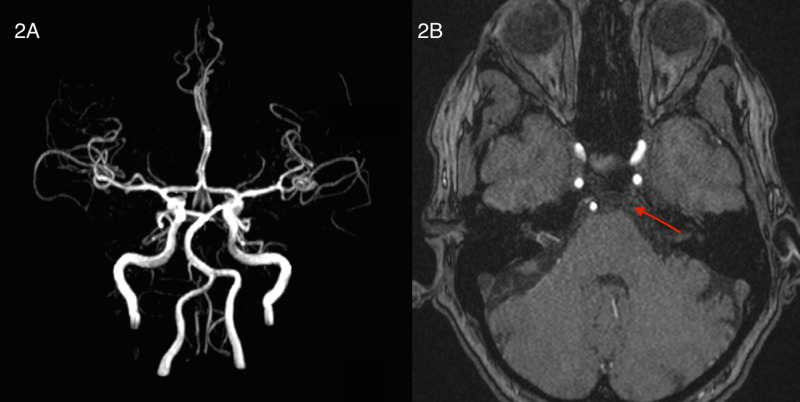
MRI. 2A: Magnetic Resonance Angiography (MRA) - Maximum Intensity Projection (MIP) images fail to reveal a left vertebral dissection. 2B: T1 weighted axial images reveal a signal void of the left V4 vertebral artery (red arrow) MRI: Magnetic Resonance Imaging

An axial T1 weighted magnetic resonance angiography (MRA) imaging study revealed a filling defect involving the left V4 segment of the distal vertebral artery from the entrance into the dura until its confluence with the basilar artery. A Magnetic Resonance Angiography (MRA)-Maximum Intensity Projection (MIP) study did not reveal the arterial dissection (Figure 2).

**Figure 3:**
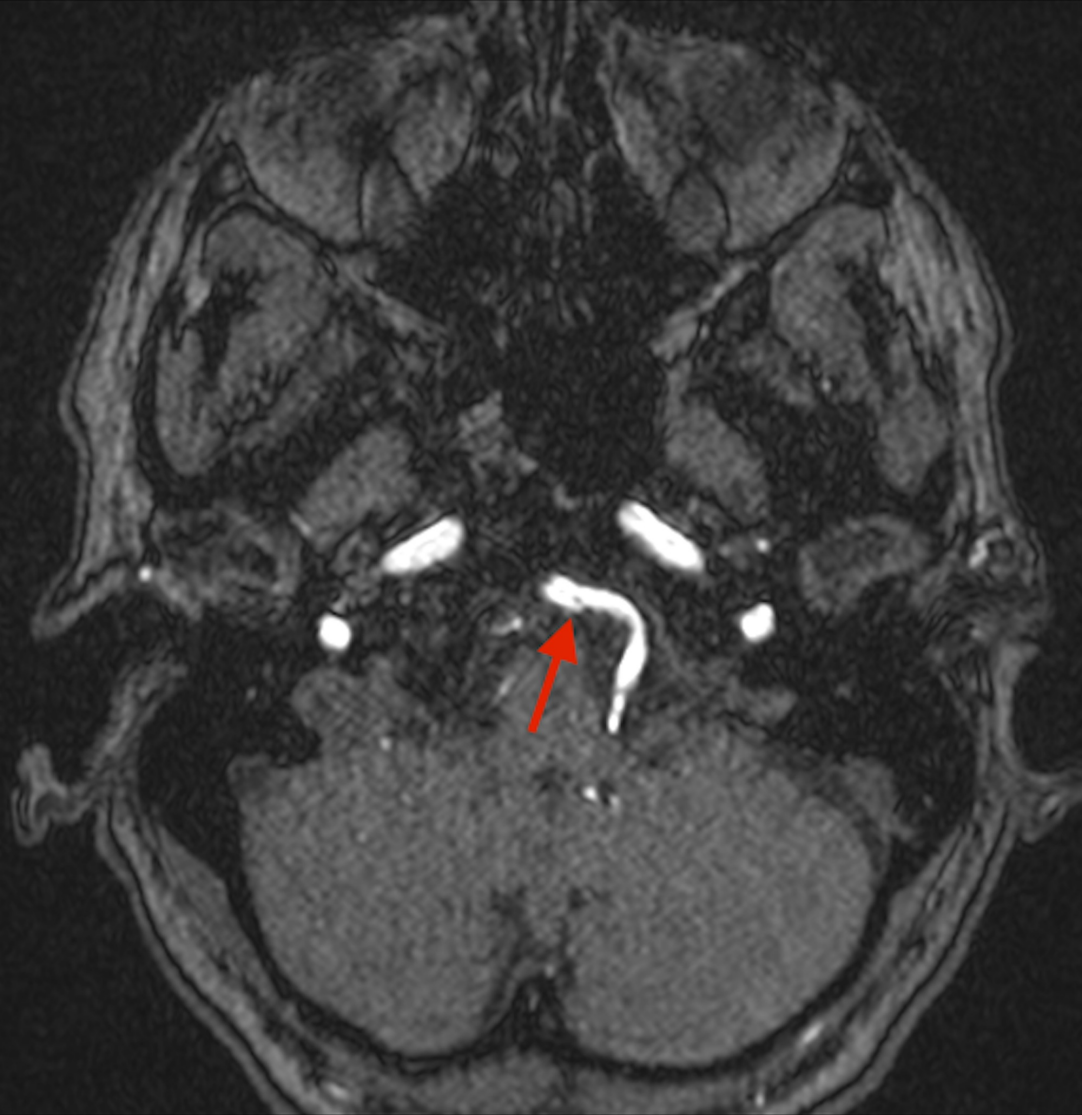
3 Dimension (D) Time of Flight (TOF) source data images reveal an intimal flap of the left V4 segment of the vertebral artery suggestive of an arterial dissection (red arrow) MRI: Magnetic Resonance Imaging

However, a 3 Dimension (D) Time of Flight (TOF) source data image shows a subtle intimal flap of an arterial dissection of the V4 segment of the left vertebral artery (Figure 3).

**Figure 4:**
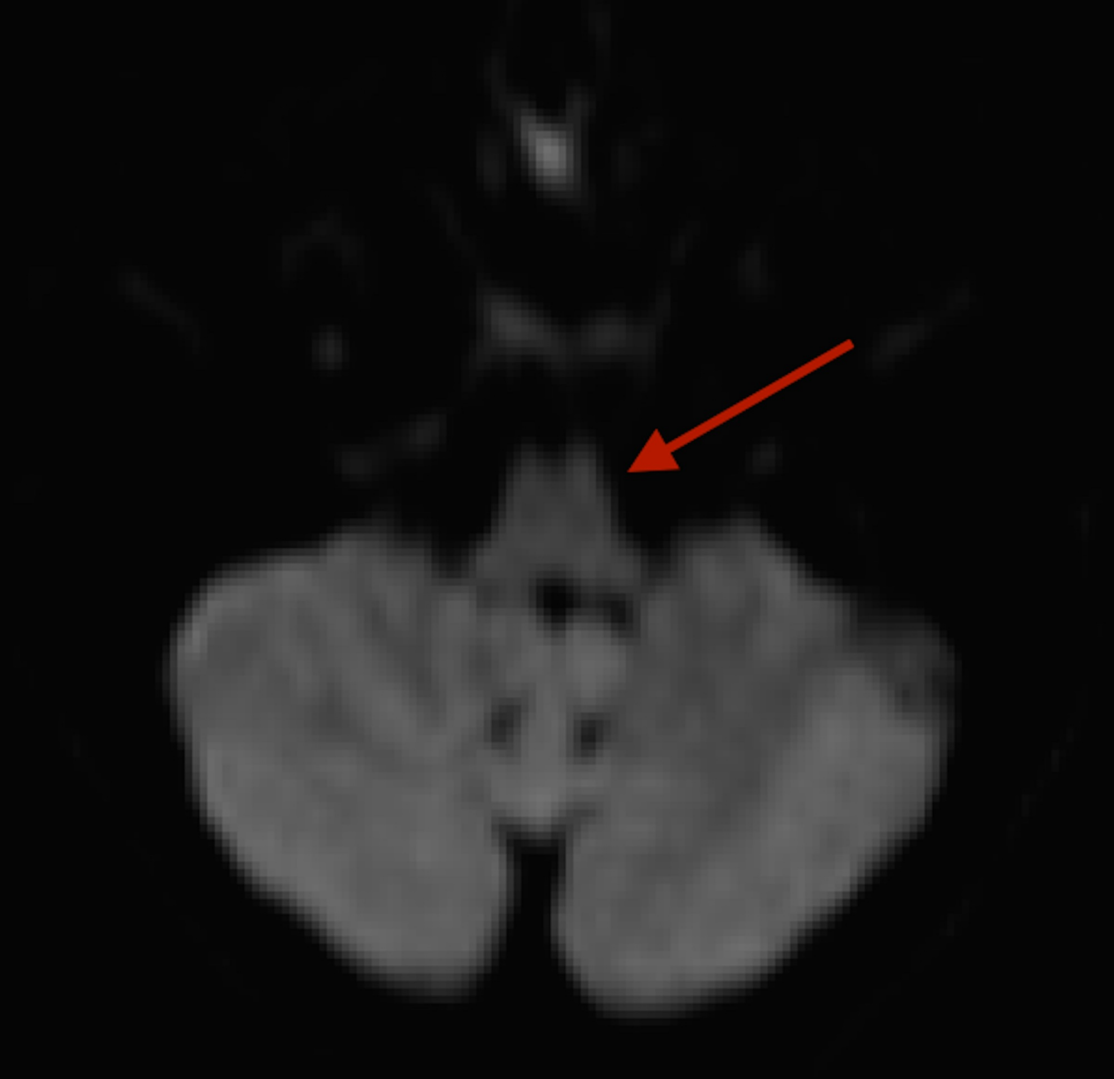
MRI Diffusion Weighted Imaging (DWI). No evidence of ischemic infarct of lower medulla (red arrow) MRI: Magnetic Resonance Imaging

An MRI of the brain failed to display a diffusion weight abnormality of the lower medulla which is concordant with the clinical examination (Figure 4).

Hence the logical assumption is to postulate that the V4 vertebral artery dissection injured the cisternal segment of the hypoglossal nerve, which happens to receives a rich blood supply from the vertebral and basilar arteries. The patient was prescribed 81 milligrams (mg) of aspirin daily and advised to wean off estrogen. She improved rapidly within two weeks; the dysarthria, dysphagia, and tongue deviation almost completely resolved. In the discussion section we will outline the neuroanatomy of the cisternal segment of the hypoglossal nerve and its blood supply, discuss the pathophysiology of arterial dissection and nerve trunk injury, and briefly outline the latest studies on the management of vertebral and carotid artery dissections.

## Discussion

**Figure 5:**
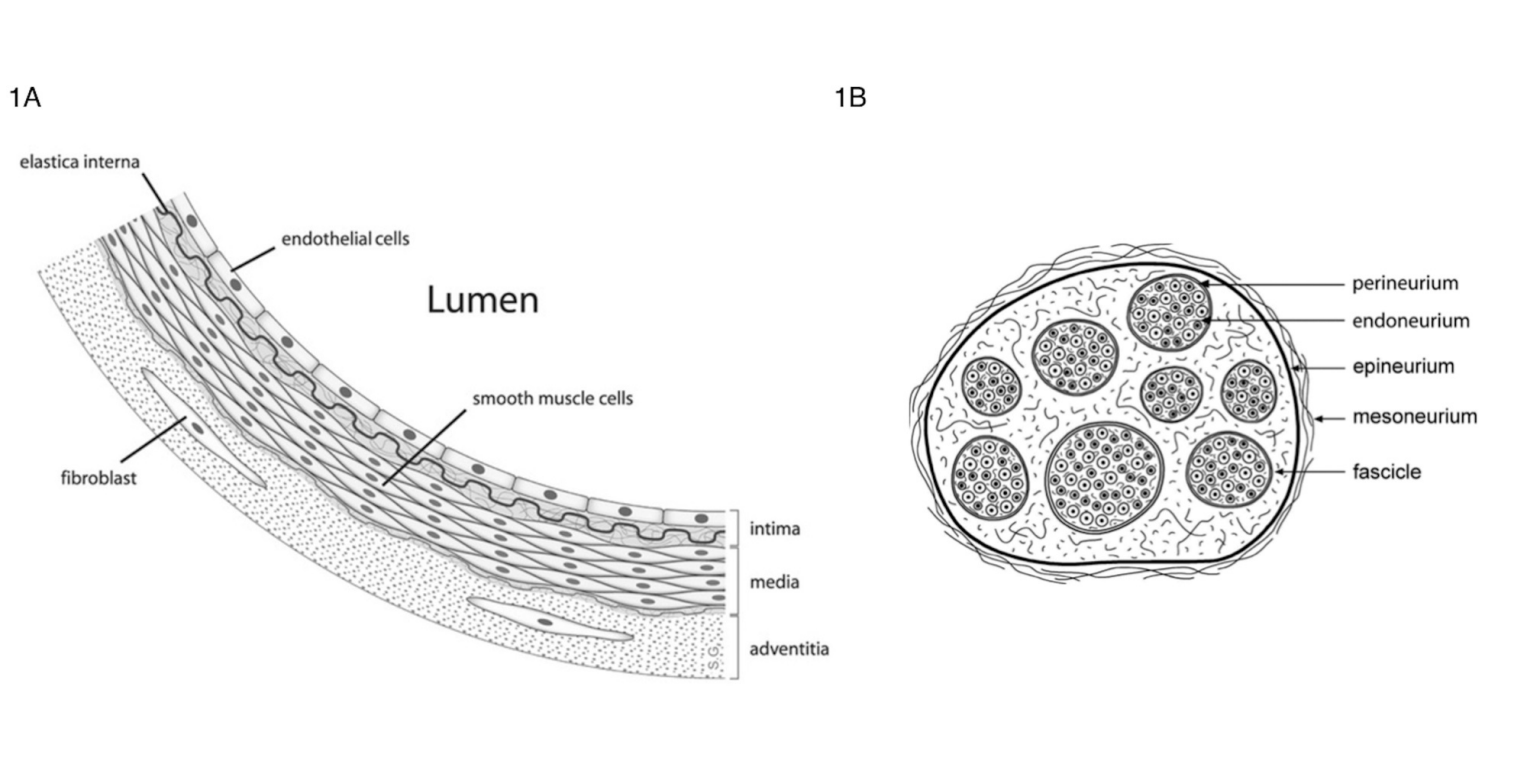
Cartoon cross sections. 1A: arterial wall. 1B: nerve trunk

As a reminder, we illustrate a cartoon of an arterial vessel wall and a cross section across a nerve trunk (Figure 5).

Arterial dissection of the internal carotid arteries leads to a subintimal tear with thrombosis and dissection. However, arterial dissection in the vertebrobasilar system leads to a tear between the tunica media and adventitia, which may lead to aneurysms, pseudoaneurysms, or subarachnoid hemorrhage, if intracranial. Defects in the internal elastic lamina induce dissection of the arterial wall [6]. In a post-mortem study of bilateral vertebral artery dissections, the thickness of the tunica media and adventitia was significantly reduced after the origin of the posterior inferior cerebellar artery. Internal elastic lamina fragmentation was also observed at multiple sites, especially in the extradural portion; V1, V2, and V3 and near the origin of the posterior inferior cerebellar artery (PICA) [7]. 

**Figure 6:**
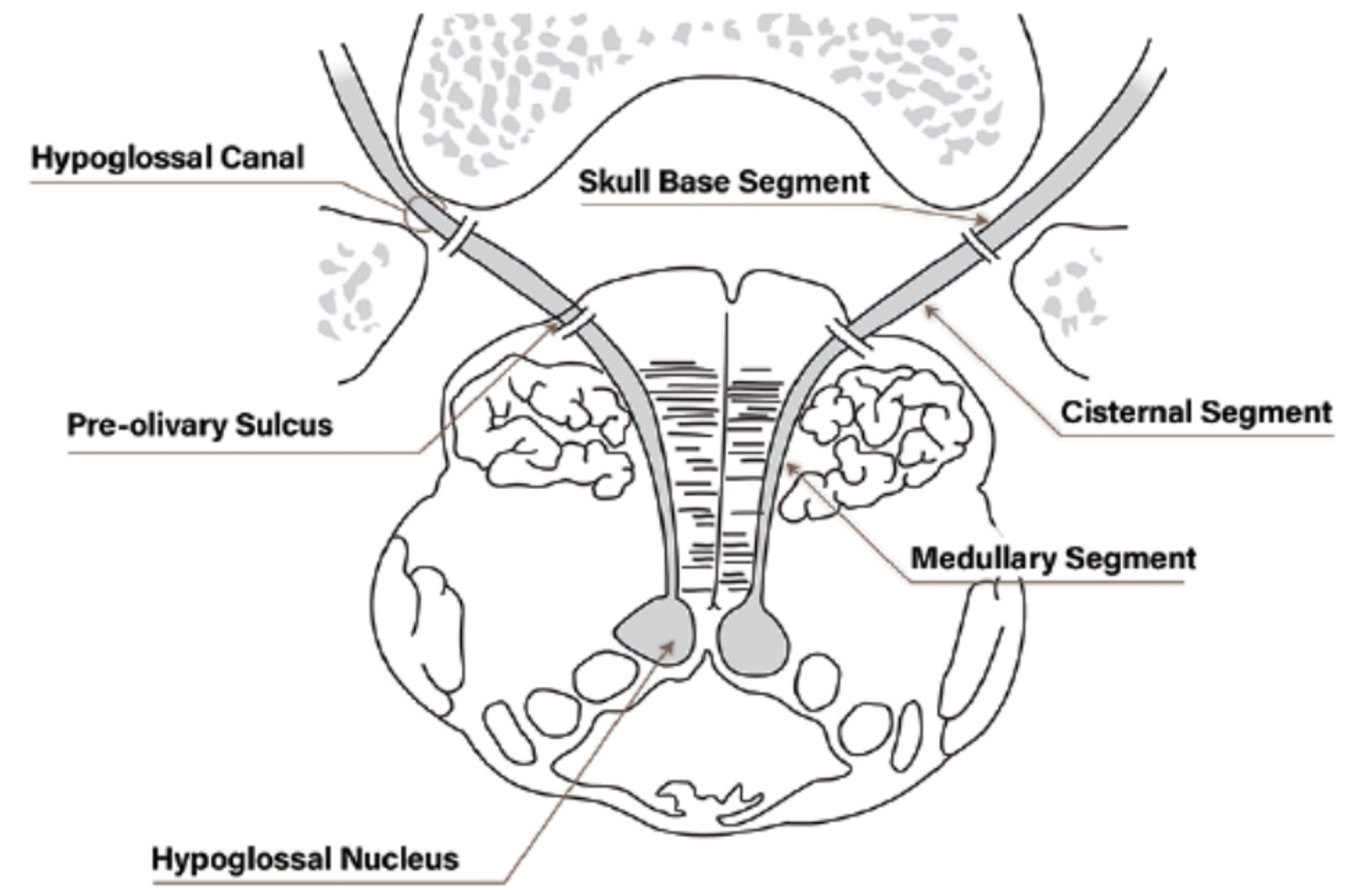
Cisternal segment of hypoglossal nerve: entrance point from the pre-olivary sulcus to the exit at the hypoglossal canal

The cisternal segment of the hypoglossal nerve consists of three to 15 long roots, which fuse to form two trunks. It extends from the pre-olivary sulcus to its entrance into the hypoglossal canal (Figure 6).

The roots of each nerve receive blood from three to five branches from the anterolateral and the lateral medullary arteries, which may arise from the perforating branches or the pontomedullary branch of the basilar artery, the vertebral artery, the anterior spinal artery, the posterior spinal artery, and the posterior inferior cerebellar artery. The main hypoglossal arteries always course along the dorsal surface of the roots of the hypoglossal nerve. The rich collateral flow may explain the excellent prognosis of fascicular ischemia to the hypoglossal nerve.

In order to understand the pathophysiology of nerve trunk neuropraxia and axontmesis, one needs to outline the topography of the blood supply to the nerve trunk and understand the mechanisms of nerve injury. There is a fine vascular net with fine vascular branches in the sheaths of nerves. There is also an intrinsic longitudinal vascular plexus that ramifies in the endoneurial and perineurial sheaths of peripheral nerve trunks and anastomoses regularly with a transverse plexus. These plexi are both intrafascicular and perifascicular. Hence there is a rich anastomotic network located both in the endoneurium and perineurium and it turns out that this network is segmental. In essence, the blood supply of the nerve trunks is segmental and derived from separate vasa nervosum. This segmentation is augmented by a rich network of longitudinal epineural and endoneurial vascular plexi that are cross-linked [8,9]. Nerve injury can be affected by either direct compression or by avulsion or ligation of the vasa nervorum (Table 1).

**Table 1:** Comparison of nerve damage semiology and pathology from direct compression and ligation of vasa nervorum: blood supply to a nerve trunk is segmental and it requires sustained damage to the epineural and endoneurial anastomotic network to impress nerve fiber degeneration

COMPRESSION/DIRECT PRESSURE	LIGATION OF BLOOD VESSEL
Pneumatic cuff	Ligation or avulsion of vasa nervorum
Ischemia of compressed segment and anoxia / local deformation of nerve	Direct devascularization
Conduction block maximal at margins of segment but prolonged compression is segmental	Conduction block variable
Requires prolonged ischemia due to dense anastamotic networks	Intrinsic longitudinal network compensates plus neovascularization
Wallerian degeneration	Axonal spheroids plus vacuolation of myelin sheath / Wallerian degeneration / collagen in endoneurium / coagulation necrosis / obliteration of Schwann tubes
Rapid recovery with segmental ischemia; requires loss of epineurial and endoneurial network for fiber degeneration	Rapid recovery with segmental ischemia; requires loss of epineurial and endoneurial network for fiber degeneration

We posit that a vertebral artery dissection obstructs the vasa nervorum of the cisternal segment of the hypoglossal nerve leading to ischemia and anoxia with conduction block. However, over time the local restitution of the dense anastomotic longitudinal plexi along with the establishment of collateral flow from the basilar artery explains the recovery of hypoglossal function. Furthermore, a neurapraxia usually arising from the conduction block may resolve within a month.

It is already established that aspirin 81-325 milligrams (mg) or clopidrogel 75 mg daily is bio-equivalent to oral anti-coagulation with warfarin for stable vertebral and carotid arterial dissections with respect to angiographic resolution and prevention of ischemic events [10,11,12,13]. A brief outline of these studies with the main outcomes is illustrated in Table 2.

**Table 2:** Bio-equivalence of anti-platelet and anti-coagulant therapy for vertebral and carotid artery dissections: brief overview with main results - no clinically significant differences between the two groups; transient ischemic attack (TIA), Cervical Artery Dissection in Stroke Study (CADISS)

STUDY	STUDY DETAILS	NUMBER OF PATIENTS	STUDY DURATION	TIA / STROKE ON ANTI-PLATELET	TIA / STROKE ON WARFARIN
Arauz et al., 2012 [10]	Aspirin vs anti-coagulation in intra- and extra-cranial vertebral artery dissection	210	21 months	only 1 case	none
Georgiadis et al., 2009 [11]	Aspirin vs anti-coagulation in carotid artery dissection: A study of 298 patients	298	3 months	2.1%	5.9%
Markus et al., 2015 [12]	CADISS-2015	250	3 months	2%	1%
Markus et al., 2019 [13]	CADISS-2019	250	12 months	4.8%	5.65%

Prior to the Cervical Artery Dissection in Stroke Study (CADISS) trial [12], the default treatment of carotid and vertebral arterial dissections was warfarin based upon empirical therapy. However, this was risky in patients with intra-dural arterial dissections due to the potential for sub-arachnoid hemorrhage [14]. Hence, the results of CADISS and the subsequent trials listed in Table 2 have greatly simplified and streamlined the treatment of vertebral and carotid arterial dissections.

## Conclusions

Our case is a rare example of fascicular nerve damage to a cranial nerve, namely the cisternal hypoglossal nerve, from a V4 vertebral artery dissection. The profuse and unique blood supply to a nerve trunk explains its uniformly good prognosis following limited duration ischemia. In addition, a conduction block from a neurapraxia is usually reversible. In addition, we briefly outline the uniformly accepted notion of anti-platelet therapy for uncomplicated vertebral artery dissections.
